# High quality draft genome sequences of *Mycoplasma agassizii* strains PS6^T^ and 723 isolated from *Gopherus* tortoises with upper respiratory tract disease

**DOI:** 10.1186/s40793-018-0315-1

**Published:** 2018-04-27

**Authors:** David Alvarez-Ponce, Chava L. Weitzman, Richard L. Tillett, Franziska C. Sandmeier, C. Richard Tracy

**Affiliations:** 1 Biology Department, University of Nevada, Reno, USA; 2 Nevada Center for Bioinformatics, University of Nevada, Reno, USA; 3 Biology Department, Colorado State University, Pueblo, USA

**Keywords:** *Mycoplasma agassizii*, Desert tortoise, Gopher tortoise, *Gopherus*, Upper respiratory tract disease (URTD), PS6^T^, ATCC 700616, 723, ATCC 700617

## Abstract

*Mycoplasma agassizii* is one of the known causative agents of upper respiratory tract disease (URTD) in Mojave desert tortoises (*Gopherus agassizii*) and in gopher tortoises (*Gopherus polyphemus*). We sequenced the genomes of *M. agassizii* strains PS6^T^ (ATCC 700616) and 723 (ATCC 700617) isolated from the upper respiratory tract of a Mojave desert tortoise and a gopher tortoise, respectively, both with signs of URTD. The PS6^T^ genome assembly was organized in eight scaffolds, had a total length of 1,274,972 bp, a G + C content of 28.43%, and contained 979 protein-coding genes, 13 pseudogenes and 35 RNA genes. The 723 genome assembly was organized in 40 scaffolds, had a total length of 1,211,209 bp, a G + C content of 28.34%, and contained 955 protein-coding genes, seven pseudogenes, and 35 RNA genes. Both genomes exhibit a very similar organization and very similar numbers of genes in each functional category. Pairs of orthologous genes encode proteins that are 93.57% identical on average. Homology searches identified a putative cytadhesin. These genomes will enable studies that will help understand the molecular bases of pathogenicity of this and other *Mycoplasma* species.

## Introduction

The genus *Mycoplasma*, within the bacterial class *Mollicutes* (*Tenericutes*), contains over one hundred species, many of which are pathogenic to vertebrates [1]. An upper respiratory tract disease has been implicated in population declines in Mojave Desert tortoises (*Gopherus agassizii*) found in the desert southwest of the United States and gopher tortoises (*Gopherus polyphemus*) inhabiting forests of the U.S. southeast [2–4]. Pathogens associated with this disease include two *Mycoplasma*, *Mycoplasma agassizii* and *Mycoplasma testudineum* [5–7]. Due to conservation concerns regarding URTD, this disease and its associated pathogens have become a topic of research interest, though our understanding of the biology and progression of URTD is lacking [8, 9]. In particular, disease in tortoises is found with varying levels of morbidity, and one hypothesis for this finding is that there is genetic variation of *M. agassizii* associated with varying levels of virulence [8]. To understand better the amount of genomic differentiation occurring between *M. agassizii* collected from different tortoise host species, and to identify markers associated with virulence, we sequenced the *M. agassizii* genome from two strains, PS6^T^ and 723. This sequencing is part of a larger project to ultimately genetically detect variation in strains and their virulence from field-cultured samples.

## Organism information

### Classification and features

*Mycoplasma agassizii* has been isolated from multiple tortoise species, and was found to be pathogenic in Mojave Desert tortoises and gopher tortoises in North America, causing URTD [5, 6, 10]. In infected North American tortoises, *M. agassizii* is most often found in the nasal passages and choana, but can also be isolated from the trachea and lungs [10]. This microbe forms a close extracellular association with the nasal epithelium of its host, and severe infections can result in lesions [11]. Infected hosts experience clinical signs of disease including nasal exudate, possibly leading to lethargic behavior and loss of appetite [5, 11].

**Fig. 1:**
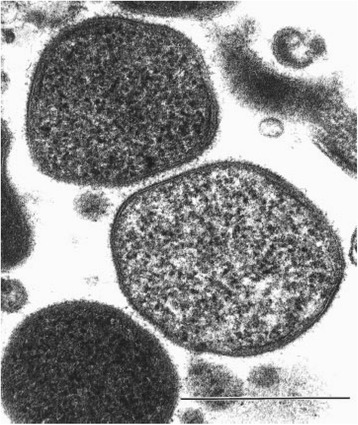
Electron micrograph of ultrathin section of *Mycoplasma agassizii* strain PS6^T^. Image from ref. [10], reproduced with permission from the publisher. Scale bar = 0.5 μm

*M. agassizii* is coccoid to pleomorphic in shape, lacks a cell wall, and has a three-layer membrane (Table 1, Fig. 1). These microbes range in size under 1 μm [10, 11] and grow in culture at an optimal temperature of 30 °C, with an extremely slow growth rate [10, 12]. Mortality of *M. agassizii* occurs at temperatures above 37 °C [12], and it retains viability after prolonged periods of cold temperatures [6, 10], indicating that body temperatures experienced by its ectothermic hosts likely affect the microbe’s success over the seasons. In an experiment to detect co-infection patterns of *M. agassizii* with its close relative *M. testudineum*, there was some indication that the two species form a facilitative relationship in a host-context-dependent manner [13]. Preliminary microbiome data suggest that the presence of *M. agassizii* is associated with a shift in the microbial community composition in Mojave and Sonoran Desert tortoises (*Gopherus morafkai*) (CLW, FCS and CRT, unpublished data).

**Table 1:** Classification and general features of *Mycoplasma agassizii*, strains PS6^T^ and 723

MIGS ID	Property	Term	Evidence code^a^
	Classification	Domain *Bacteria*	TAS [38]
		Phylum *Firmicutes*	TAS [39]
		Class *Mollicutes*	TAS [40]
		Order *Mycoplasmatales*	TAS [41, 42]
		Family *Mycoplasmataceae*	TAS [42]
		Genus *Mycoplasma*	TAS [10]
		Species *Mycoplasma agassizii*	TAS [10]
		Strains PS6^T^ and 723	TAS [5, 6, 10, 20]
	Gram stain	Negative	NAS
	Cell shape	Coccoid to pleomorphic	TAS [10]
	Motility	Non-motile	TAS [10]
	Sporulation	Nonspore-forming	NAS
	Temperature range	Not reported	
	Optimum temperature	30 °C	TAS [10]
	pH range; Optimum	Not reported	
	Carbon source	Glucose	TAS [10]
MIGS-6	Habitat	Tortoise respiratory tract	TAS [10]
MIGS-6.3	Salinity	Not reported	
MIGS-22	Oxygen requirement	Aerobic	TAS [10]
MIGS-15	Biotic relationship	Symbiont	TAS [11]
MIGS-14	Pathogenicity	Pathogenic	TAS [5, 6]
MIGS-4	Geographic location	North America	TAS [6, 10]
MIGS-5	Sample collection	1991 (PS6^T^), 1992 (723)	TAS [43]
MIGS-4.1	Latitude	Approx.: 36 N (PS6^T^), 26.4 N (723)	TAS [6, 10]
MIGS-4.2	Longitude	Approx.: 115 W (PS6^T^), 82.1 W (723)	TAS [6, 10]
MIGS-4.4	Altitude	Approx.: 800 m (PS6^T^), 0 m (723)	TAS [6, 10]

*IDA* Inferred from Direct Assay, *TAS* Traceable Author Statement (i.e., a direct report exists in the literature), *NAS* Non-traceable Author Statement (i.e., not directly observed for the living, isolated sample, but based on a generally accepted property for the species, or anecdotal evidence). These evidence codes are from the Gene Ontology project [44]

^a^Evidence codes

The strains of *M. agassizii* that we have sequenced were isolated from two host species. Strain PS6^T^ was isolated from the upper respiratory tract of a Mojave Desert tortoise in the Las Vegas Valley, Nevada, USA [10], while strain 723 was obtained from an ill gopher tortoise in Sanibel Island, Florida, USA [6]. Strains were cultured in SP4 broth, and have been used in experiments to demonstrate their pathogenic effects on their tortoise hosts [5, 6].

**Fig. 2:**
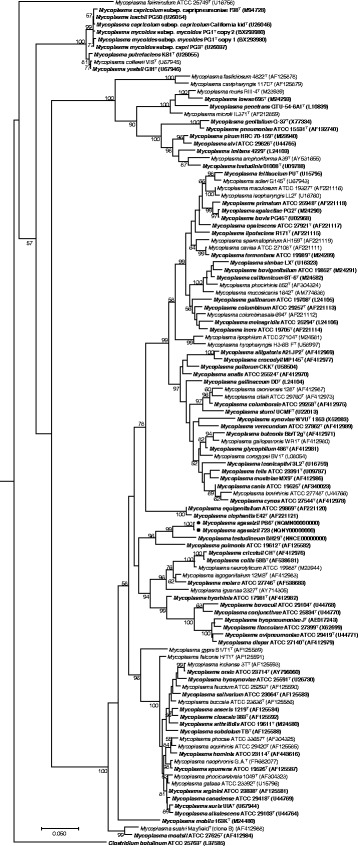
Phylogenetic tree of the *Mycoplasma* genus based on 16S rRNA gene sequences showing the phylogenetic position of *M. agassizii* PS6^T^ and 723 (●). All 16S sequences from the *Mycoplasma* genus were obtained from the SILVA database [14]. Only sequences in the ‘The All-Species Living Tree’ Project (LTP), release 128, were retained. This dataset only contains sequences from type strains, designated with a superscripted “T”. *Clostridium botulinum* strain ATCC 25763 was also included in the dataset as outgroup. Sequences were aligned using MUSCLE 3.8.31 [15]. A phylogenetic tree was obtained using the maximum likelihood method implemented in MEGA7 [16], with 1000 bootstrap replicates. Species with available genomes at the NCBI Genomes database or the Genomes Online Database are represented in bold face. GenBank accession numbers are shown in parentheses. Bootstrap support values above 50% are represented. The scale bar represents a divergence of 0.05 nucleotide substitutions per nucleotide position

To determine the placement of *M. agassizii* in the mycoplasmal phylogeny, all 16S rRNA gene sequences from the type strains of *Mycoplasma* species were obtained from the SILVA database [14] and aligned using MUSCLE 3.8.31 [15], and a phylogenetic tree was constructed using the maximum likelihood method implemented in MEGA7 [16] (Fig. 2). Consistent with prior results [17, 18], *M. testudineum* is a sister group of *M. agassizii* in the resultant tree, and the *M. agassizii*/*M. testudineum* clade is a sister group of *Mycoplasma pulmonis*, the agent of murine respiratory mycoplasmosis. All three species fall within the hominis group of *Mycoplasma* (see ref. [19] for group definitions). The 16S rRNA gene sequence from *M. agassizii*, strain PS6^T^, is 99.8, 93.2 and 89.2% identical to those of *M. agassizii* strain 723, *M. testudineum* strain BH29^T^, and *M. pulmonis* strain PG34^T^, respectively.

## Genome sequencing information

### Genome project history

Two strains of *M. agassizii* were selected for sequencing, strains PS6^T^ and 723, both isolated from tortoises with signs of URTD [5, 6, 10, 20]. Sequencing was conducted in October 2016. The Whole Genome Shotgun projects were deposited at DDBJ/ENA/GenBank under the accession numbers NQMN00000000 (strain PS6^T^) and NQNY00000000 (strain 723). The versions described in this paper are the first versions. A summary of the information of both projects in compliance with MIGS version 2.0 [21] is shown in Table 2.

**Table 2:** Project information

MIGS ID	Property	Term
MIGS-31	Finishing quality	High quality drafts
MIGS-28	Libraries used	Illumina Nextera XT
MIGS-29	Sequencing platforms	Illumina NextSeq500
MIGS-31.2	Fold coverage	38.51 (strain PS6^T^); 37.73 (strain 723)
MIGS-30	Assemblers	SPAdes 3.10.1
MIGS-32	Gene calling method	NCBI Prokaryotic Genome Annotation Pipeline 4.2
	Locus Tag	CJF60 (strain PS6^T^); CJJ23 (strain 723)
	GenBank ID	NQMN00000000 (strain PS6^T^); NQNY00000000 (strain 723)
	GenBank Date of Release	August 28, 2017 (strain PS6^T^); August 29, 2017 (strain 723)
	GOLD ID	–
	BIOPROJECT	PRJNA397947 (strain PS6^T^); PRJNA398096 (strain 723)
MIGS-13	Source Material Identifier	ATCC 700616 (strain PS6^T^); ATCC 700617 (strain 723)
	Project relevance	Animal parasite

### Growth conditions and genomic DNA preparation

Freeze-dried *M. agassizii* strains were obtained from the ATCC in March 2011 (strain PS6^T^) and May 2016 (strain 723). Strain PS6^T^ was cultured on SP4 media and re-pelleted in-lab prior to DNA extraction. Genomic DNA was extracted using the Qiagen DNeasy Blood and Tissue Kit protocol for Gram-negative bacteria and eluted with water. Extracted DNA was quantified on a Qiagen QIAxpert system and with Picogreen analysis.

### Genome sequencing and assembly

Genome sequencing was conducted using the Illumina Nextera XT DNA Library Preparation Kit (Illumina, Inc., San Diego, USA) with the Illumina NextSeq500 platform (150 bp, paired-end) and 2 ng of starting genomic DNA at the Nevada Genomics Center (University of Nevada, Reno). Sequencing was performed in multiplex with multiple samples, using dual index sequences from the Illumina Nextera XT Index Kit, v2 (PS6 indices: index 1 N702, index 2 S510; 723 indices: index 1 N702, index 2 S511). A total of 349,251 and 332,967 read pairs were obtained for strains PS6^T^ and 723, respectively. Using Trimmomatic, version 0.36 [22], reads were trimmed to remove Nextera adapter sequences and low quality nucleotides from either end (average Phred score Q ≤ 5, four bp sliding window), and sequences trimmed to < 35 bp were removed. After trimming, 330,351 (PS6^T^) and 305,002 (723) read pairs, and 16,438 (PS6^T^) and 25,017 (723) single-reads (the pairs of which were removed) remained. De novo genome assembly was performed using SPAdes 3.10.1 [23], using as inputs the trimmed paired reads, and the trimmed single reads (assembly k-mer sizes 21, 33, 55, and 77, with read error-correction enabled and ‘--careful’ mode mismatch correction). After removing scaffolds of less than 500 bp, the final assemblies consisted of 8 (PS6^T^) and 40 (723) scaffolds with a total length of 1,274,972 bp (PS6^T^) and 1,211,209 bp (723), an average length of 159,372 bp (PS6^T^) and 30,280 bp (723), and an N50 of 654,010 bp (PS6^T^) and 56,701 bp (723). The coverage was 38.51× for the PS6^T^ assembly and 37.73× for the 723 assembly.

### Genome annotation

Gene prediction was carried out using the NCBI Prokaryotic Genome Annotation Pipeline 4.2 [24]. For each predicted protein, (*i*) families were identified using the Pfam 31.0 [25] batch search tool (“gathering threshold” option), (*ii*) Clusters of Orthologous Groups categories were assigned using eggNOG-mapper [26] based on eggNOG 4.5.1 data [27], (*iii*) signal peptides were identified using the SignalP server 4.1 [28], and (*iv*) transmembrane helices were inferred using the TMHMM server v. 2.0 [29]. CRISPR repeats were identified using PGAP and CRISPRFinder [30].

## Genome properties

The properties of both draft genomes are summarized in Table 3. The final assembly for strain PS6^T^ consisted of 8 scaffolds, with a total length of 1,274,972 bp, and a G + C content of 28.43%. PGAP [24] identified a total of 979 protein-coding genes, 13 pseudogenes, and 35 RNA genes. The assembly for strain 723 consisted of 40 scaffolds, with a total length of 1,211,209 bp, and a G + C content of 28.34%. A total of 955 protein-coding genes, 7 pseudogenes, and 35 RNA genes were identified. In both cases, the identified RNA genes include 3 rRNAs (one 5S, one 16S and one 23S), 3 ncRNAs and 29 tRNAs. PGAP identified no CRISPR repeats in any of the two genomes, and CRISPRFinder [30] identified 6 “questionable” repeats in the PS6^T^ genome and one “questionable” repeat in the 723 genome, but no “confirmed” repeats. The numbers of protein-coding genes in each COG category [31] were similar for both strains, and are summarized in Table 4.

**Table 3:** Genome statistics

Attribute	Strain PS6^T^		Strain 723	
	Value	% of Total	Value	% of Total
Genome size (bp)	1,274,972	100.00	1,211,209	100.00
DNA coding (bp)	1124,547^a^	88.20^c^	1072,218^a^	88.52^c^
DNA G + C (bp)	362,520	28.43^c^	343,241	28.34^c^
DNA scaffolds	8	100.00	40	100.00
Total genes	1027	100.00	997	100.00
Protein coding genes	979	95.33^d^	955	95.79^d^
RNA genes	35	3.41^d^	35	3.51^d^
Pseudo genes	13	1.27^d^	7	0.70^d^
Genes in internal clusters	–	–	–	–
Genes with function prediction	467^b^	47.70^e^	301^b^	31.52^e^
Genes assigned to COGs	581	59.35^e^	577	60.42^e^
Genes with Pfam domains	608	62.10^e^	607	63.56^e^
Genes with signal peptides	160	16.34^e^	150	15.71^e^
Genes with transmembrane helices	294	30.03^e^	288	30.16^e^
CRISPR repeats	0	–	0	–

^a^Protein-coding sequences, not including stop codons

^b^Proteins not annotated as “hypothetical protein” by PGAP

^c^Relative to genome size

^d^Relative to total number of genes

^e^Relative to protein-coding genes

**Table 4:** Number of genes associated with general COG functional categories

Code^a^	Strain PS6^T^		Strain 723		Description
	Value^b^	%age	Value^b^	%age	
J	101	10.32	101	10.58	Translation, ribosomal structure and biogenesis
A	0	0.00	0	0.00	RNA processing and modification
K	21	2.15	20	2.09	Transcription
L	66	6.74	60	6.28	Replication, recombination and repair
B	0	0.00	0	0.00	Chromatin structure and dynamics
D	4	0.41	5	0.52	Cell cycle control, Cell division, chromosome partitioning
V	33	3.37	29	3.04	Defense mechanisms
T	5	0.51	5	0.52	Signal transduction mechanisms
M	10	1.02	10	1.05	Cell wall/membrane biogenesis
N	0	0.00	0	0.00	Cell motility
U	11	1.12	9	0.94	Intracellular trafficking and secretion
O	28	2.86	31	3.25	Posttranslational modification, protein turnover, chaperones
C	34	3.47	34	3.56	Energy production and conversion
G	72	7.35	74	7.75	Carbohydrate transport and metabolism
E	27	2.76	26	2.72	Amino acid transport and metabolism
F	25	2.55	25	2.62	Nucleotide transport and metabolism
H	13	1.33	13	1.36	Coenzyme transport and metabolism
I	9	0.92	9	0.94	Lipid transport and metabolism
P	35	3.58	36	3.77	Inorganic ion transport and metabolism
Q	1	0.10	1	0.10	Secondary metabolites biosynthesis, transport and catabolism
R	0	0.00	0	0.00	General function prediction only
S	92	9.40	93	9.74	Function unknown
–	398	40.65	378	39.58	Not in COGs

Percentages are based on the total number of protein coding genes in the genome

^a^COG category code

^b^Number of genes in the category

## Insights from the genome sequence

The small genome size and low G + C content of both *M. agassizii* genomes described here are consistent with those of other *Mycoplasma* genomes sequenced [18, 32, 33]. However, the *M. agassizii* genomes are significantly larger than the genome of *M. testudineum*, strain BH29^T^ (960,895 bp, 788 protein-coding genes; ref. [18]). The difference in the genome size of both sister species might account for the fact that *M. agassizii* is associated with URTD, whereas the link between *M. testudineum* and URTD is less clear [13]; i.e., genes present in *M. agassizii* but not in *M. testudineum* might be responsible for pathogenicity.

**Fig. 3:**
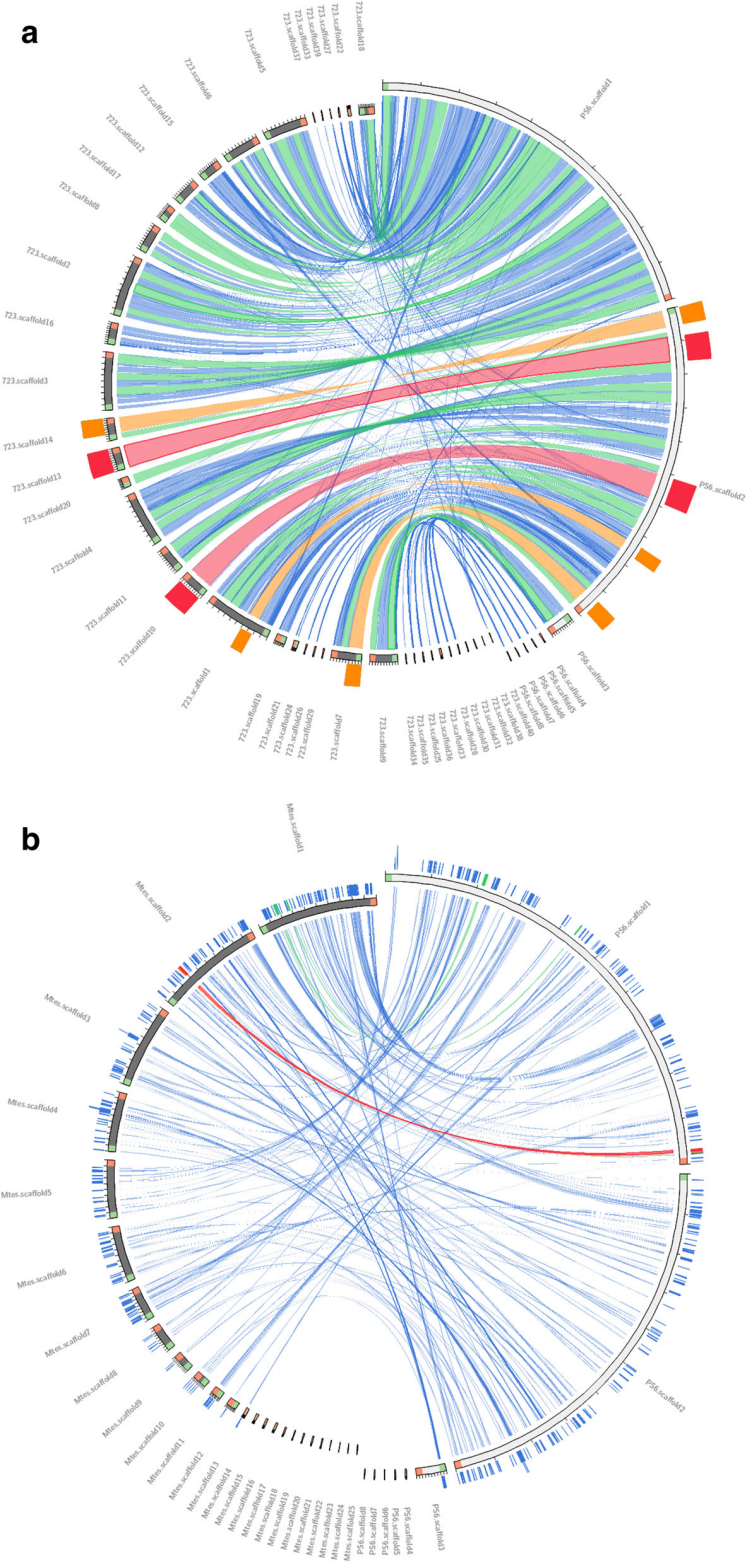
Comparison of the genomes of *M. agassizii* strains PS6^T^ and 723 (**a**), and *M. agassizii* strain PS6^T^ and *M. testudineum* strain BH29^T^ (**b**). The figure was generated using Circoletto 07.09.16 [45], a web interface for Circos [46]. The relative order of scaffolds is unknown. For strain PS6^T^, scaffolds are sorted by size

In spite of the fact that the two *M. agassizii* strains sequenced here were obtained from geographically distant locations (the Mojave Desert and Sanibel Island) and from different tortoise species (*G. agassizii* and *G. polyphemus*; refs. [5, 6, 10, 20]), the two genomes are very similar, exhibiting very similar sizes, numbers of genes (Table 3), functional composition (Table 4), and a high degree of synteny (Fig. 3a). A best-reciprocal-hit approach (based on BLASTP searches, *E*-value ≤10^− 10^) identified 828 pairs of putative orthologs within both genomes. The sequences of proteins encoded by pairs of orthologous genes were aligned using ProbCons version 1.12 [34], and were 93.57% identical on average (median: 96.84%). In contrast, comparison of the genomes of *M. agassizii* strain PS6^T^ and *M. testudineum* strain BH29^T^ [18] revealed much less synteny (Fig. 3b) and protein identity (average: 54.78%, median: 54.71%).

**Fig. 4:**
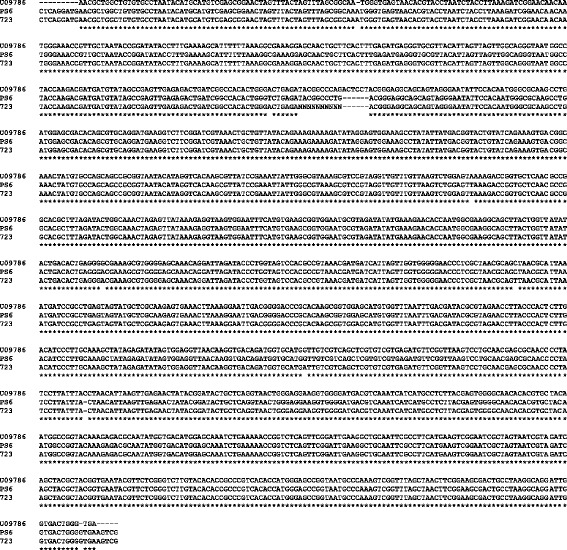
Comparison of the 16S rRNA gene sequences generated by Brown et al. [20] (*M. agassizii* strain PS6^T^; GenBank accession: U09786) and in our study (*M. agassizii* strains PS6^T^ and 723). Asterisks represent identical sites

The 16S rRNA gene sequences of *M. agassizii*, strains PS6^T^ and 723, differed at 3 nucleotide positions (Fig. 4). Surprisingly, our 16S sequence for strain PS6^T^ and that obtained by Brown et al. (also for strain PS6^T^; ref. [20]) exhibit 8 differences (4 point differences and 4 indels; Fig. 4). These differences may represent mutations accumulated since the isolation of the strain, or sequencing errors.

To initiate pathogenesis, *Mycoplasma* cells usually require adhering to the host mucosa. Adhesion mechanisms are relatively well understood in *Mycoplasma pneumoniae* and its close relatives, but poorly understood in other *Mycoplasma* species [35]. In a prior study, we searched all available *Mycoplasma* genomic data (nr database, including the genome of *M. testudineum* BH29^T^) for homologs of *M. pneumoniae* cytadhesins P1, P30, P65, P40 and P90 and cytadhesin accessory proteins hmw1, hmw2 and hmw3, finding homologs only in species closely related to *M. pneumoniae* (*Mycoplasma genitalium*, *Mycoplasma gallisepticum*, *Mycoplasma pirum*, *Mycoplasma alvi*, *Mycoplasma imitans*, and *Mycoplasma testudinis*) [18]. Here, we expanded these analyses (BLASTP and TBLASTN searches; *E* < 10^− 5^ and low-complexity regions filtering) to the two *M. agassizii* proteomes/genomes, also with negative results. In addition, none of the predicted *M. agassizii* proteins exhibit any of the Pfam domains present in the *M. pneumoniae* (domains “CytadhesinP1”, “Adhesin_P1”, “Cytadhesin_P30”, “MgpC” and “EAGR_box”). This could be attributed either to (*i*) *M. pneumoniae* adhesion proteins being specific to this species and its close relatives, or (*ii*) adhesion proteins evolving very fast, perhaps due to co-evolutionary races, precluding detection of homologs in distantly related species. The first possibility is supported by the fact that *M. pulmonis*, the most closely related known species to the *M. agassizii*/*M. testudineum* clade, exhibits adhesion mechanisms different from *M. pneumoniae*, lacking an attachment organelle [36]. In support of the second scenario, our analysis of orthologous sequences revealed poor protein conservation among the sister groups *M. agassizii* and *M. testudineum*.

We repeated our similarity searches using as query a list of known *Mycoplasma* adhesins, which we obtained by searching the text “*Mycoplasma* adhesin” in the UniProt database [37]. Our prior searches against the *M. testudineum* BH29^T^ proteome/genome failed to detect any significant hits. In the current study, we detected significant similarity between a *Mycoplasma mobile* protein annotated as a “Truncated adhesin protein” (UniProt ID: Q8L3E5_9MOLU) and the proteins CJF60_05070 (strain PS6^T^, 3308 amino acids) and CJJ23_03020 (strain 723, also 3308 amino acids) of *M. agassizii*. CJF60_05070 and CJJ23_03020 are 92% identical. The C-terminal part of the *M. mobile* protein exhibits homology to three regions of the *M. agassizii* proteins (positions 958–1261, 1296–1597 and 1717–1924 of CJF60_05070; positions 956–1259, 1294–1595 and 1715–1922 of CJJ23_03020). A BLASTP search using CJF60_05070 as query sequence against the nr database identified a total of 17 hits, including three adhesion proteins (Table 5). Of note, the first hit is a *M. testudineum* protein (34%), which was not detected in our prior analyses [18]. Equivalent results were obtained using the CJJ23_03020 protein sequence as query (data not shown). The TMHMM server v. 2.0 [29] predicted both CJF60_05070 and CJJ23_03020 to contain a transmembrane domain at the N-terminal part of the protein (positions 7–29), and most of the protein (positions 30–3308) to be extracellular. Taken together, these observations point to these proteins as potential *M. agassizii* adhesins.

**Table 5:** Results of a BLASTP search using CJF60_05070 as query against the nr database

Accession	Description	Total score	Query cover	*E*-value	Identity
WP_094254640.1	hypothetical protein [*Mycoplasma testudineum*]	1254	98%	0.0	34%
CAC13384.1	unknown; predicted coding region [*Mycoplasma pulmonis*]	683	98%	0.0	27%
WP_041363975.1	hypothetical protein [*Mycoplasma pulmonis*]	682	98%	0.0	26%
WP_011264623.1	Gli349 adhesion and gliding protein [*Mycoplasma mobile*]	310	67%	10^−80^	25%
CCY45197.1	fNIP repeat-containing protein [*Clostridium sp*. CAG:1193]	105	2%	2 × 10^−4^	38%
WP_015135277.1	hypothetical protein [*Leptolyngbya sp*. PCC 7376]	215	5%	3 × 10^−4^	34%
AET68682.1	conserved repeat protein [*Desulfosporosinus orientis* DSM 765]	58.5	3%	3 × 10^−4^	38%
OPH56032.1	hypothetical protein BC351_29535 [*Paenibacillus ferrarius*]	105	5%	4 × 10^−4^	35%
KRK80309.1	adhesion exoprotein [*Lactobacillus nodensis* DSM 19682 = JCM 14932 = NBRC 107160]	57.8	2%	5 × 10^−4^	40%
WP_081776155.1	hypothetical protein [*Lactobacillus nodensis*]	57.4	2%	6 × 10^−4^	40%
CCY44912.1	fNIP repeat-containing protein [*Clostridium sp*. CAG:1193]	55.1	3%	6 × 10^−4^	35%
WP_057878036.1	hypothetical protein [*Lactobacillus paucivorans*]	53.5	4%	0.010	30%
WP_066545473.1	hypothetical protein [*Caryophanon tenue*]	53.1	5%	0.012	29%
WP_081780332.1	hypothetical protein [*Porphyromonas uenonis*]	97.8	2%	0.150	37%
BAB92076.1	truncated adhesin protein [*Mycoplasma mobile*]	47.4	9%	0.770	24%

## Conclusions

We have obtained draft genome sequences for *M. agassizii*, strains PS6^T^ and 723, both isolated from tortoises of the genus *Gopherus* with URTD. Both genomes exhibited a very small size and low G + C content, which is typical from *Mycoplasma* genomes. The two assemblies were very similar, in terms of synteny and protein sequences, in spite of the fact that they were obtained from different hosts and geographical locations. We identified a putative cytadhesin in both genomes. The new genomes will facilitate future studies that will help understand the molecular bases of pathogenicity of this and other *Mycoplasma* species.

## Abbreviations

ATCC: American Type Culture Collection
BLAST: Basic local alignment search tool
COG: Clusters of Orthologous Groups
MIGS: Minimum information on the genome sequence
MRM: Murine respiratory mycoplasmosis
NCBI: National Center for Biotechnology Information
PGAP: Prokaryotic Genome Annotation Pipeline
URTD: Upper respiratory tract disease

## References

[1] Brown DR (2002). Mycoplasmosis and immunity of fish and reptiles. Front Biosci.

[2] Seigel RA, Smith RB, Seigel NA (2003). Swine flu or 1918 pandemic? Upper respiratory tract disease and the sudden mortality of gopher tortoises (Gopherus polyphemus) on a protected habitat in Florida. J Herpetol.

[3] McCoy ED, Mushinsky HR, Lindzey J (2007). Conservation strategies and emergent diseases: the case of upper respiratory tract disease in the gopher tortoise. Chelonian Conserv Biol.

[4] Desert Tortoise Recovery Team. Desert tortoise (Mojave population): recovery plan. Portland: US Fish and Wildlife Service; 1994.

[5] Brown MB, Schumacher IM, Klein PA, Harris K, Correll T, Jacobson ER (1994). Mycoplasma agassizii causes upper respiratory tract disease in the desert tortoise. Infect Immun.

[6] Brown M, McLaughlin G, Klein P, Crenshaw B, Schumacher I, Brown D, Jacobson E (1999). Upper respiratory tract disease in the gopher tortoise is caused by mycoplasma agassizii. J Clin Microbiol.

[7] Brown D, Merritt J, Jacobson E, Klein P, Tully J, Brown M (2004). Mycoplasma testudineum sp. nov., from a desert tortoise (Gopherus agassizii) with upper respiratory tract disease. Int J Syst Evol Microbiol.

[8] Sandmeier FC, Tracy CR, Hunter K (2009). Upper respiratory tract disease (URTD) as a threat to desert tortoise populations: a reevaluation. Biol Conserv.

[9] Diemer Berish JE, Wendland LD, Kiltie RA, Garrison EP, Gates CA (2010). Effects of mycoplasmal upper respiratory tract disease on morbidity and mortality of gopher tortoises in northern and Central Florida. J Wildl Dis.

[10] Brown M, Brown D, Klein P, McLaughlin G, Schumacher IM, Jacobson E, Adams H, Tully J (2001). Mycoplasma agassizii sp. nov., isolated from the upper respiratory tract of the desert tortoise (Gopherus agassizii) and the gopher tortoise (Gopherus polyphemus). Int J Syst Evol Microbiol.

[11] Jacobson ER, Gaskin J, Brown M, Harris R, Gardiner C, LaPointe J, Adams H, Reggiardo C (1991). Chronic upper respiratory tract disease of free-ranging desert tortoises (Xerobates agassizii). J Wildl Dis.

[12] Mohammadpour HA (2011). Mycoplasma agassizii infections in the desert tortoise (*Gopherus agassizii*). PhD dissertation.

[13] Weitzman CL, Gov R, Sandmeier FC, Snyder SJ, Tracy CR (2017). Co-infection does not predict disease in Gopherus tortoises. Royal Soc Open Sci.

[14] Quast C, Pruesse E, Yilmaz P, Gerken J, Schweer T, Yarza P, Peplies J, Glockner FO (2013). The SILVA ribosomal RNA gene database project: improved data processing and web-based tools. Nucleic Acids Res.

[15] Edgar RC (2004). MUSCLE: multiple sequence alignment with high accuracy and high throughput. Nucleic Acids Res.

[16] Kumar S, Stecher G, Tamura K (2016). MEGA7: molecular evolutionary genetics analysis version 7.0 for bigger datasets. Mol Biol Evol.

[17] Volokhov DV, Simonyan V, Davidson MK, Chizhikov VE (2012). RNA polymerase beta subunit (rpoB) gene and the 16S–23S rRNA intergenic transcribed spacer region (ITS) as complementary molecular markers in addition to the 16S rRNA gene for phylogenetic analysis and identification of the species of the family Mycoplasmataceae. Mol Phylogenet Evol.

[18] Weitzman CL, Tillett RL, Sandmeier FC, Tracy CR, Alvarez-Ponce D (2018). High quality draft genome sequence of Mycoplasma testudineum strain BH29T, isolated from the respiratory tract of a desert tortoise. Stand Genomic Sci.

[19] Weisburg W, Tully J, Rose D, Petzel J, Oyaizu H, Yang D, Mandelco L, Sechrest J, Lawrence T, Van Etten J (1989). A phylogenetic analysis of the mycoplasmas: basis for their classification. J Bacteriol.

[20] Brown D, Crenshaw B, McLaughlin G, Schumacher I, McKenna C, Klein P, Jacobson E, Brown M (1995). Taxonomic analysis of the tortoise mycoplasmas mycoplasma agassizii and mycoplasma testudinis by 16S rRNA gene sequence comparison. Int J Syst Evol Microbiol.

[21] Field D, Garrity G, Gray T, Morrison N, Selengut J, Sterk P, Tatusova T, Thomson N, Allen MJ, Angiuoli SV (2008). The minimum information about a genome sequence (MIGS) specification. Nat Biotechnol.

[22] Bolger AM, Lohse M, Usadel B (2014). Trimmomatic: a flexible trimmer for Illumina sequence data. Bioinformatics.

[23] Bankevich A, Nurk S, Antipov D, Gurevich AA, Dvorkin M, Kulikov AS, Lesin VM, Nikolenko SI, Pham S, Prjibelski AD (2012). SPAdes: a new genome assembly algorithm and its applications to single-cell sequencing. J Comp Biol.

[24] Tatusova T, DiCuccio M, Badretdin A, Chetvernin V, Nawrocki EP, Zaslavsky L, Lomsadze A, Pruitt KD, Borodovsky M, Ostell J (2016). NCBI prokaryotic genome annotation pipeline. Nucleic Acids Res.

[25] Finn RD, Coggill P, Eberhardt RY, Eddy SR, Mistry J, Mitchell AL, Potter SC, Punta M, Qureshi M, Sangrador-Vegas A (2016). The Pfam protein families database: towards a more sustainable future. Nucleic Acids Res.

[26] Huerta-Cepas J, Forslund K, Pedro Coelho L, Szklarczyk D, Juhl Jensen L, von Mering C, Bork P (2017). Fast genome-wide functional annotation through orthology assignment by eggNOG-mapper. Mol Biol Evol.

[27] Huerta-Cepas J, Szklarczyk D, Forslund K, Cook H, Heller D, Walter MC, Rattei T, Mende DR, Sunagawa S, Kuhn M (2015). eggNOG 4.5: a hierarchical orthology framework with improved functional annotations for eukaryotic, prokaryotic and viral sequences. Nucleic Acids Res.

[28] Petersen TN, Brunak S, von Heijne G, Nielsen H (2011). SignalP 4.0: discriminating signal peptides from transmembrane regions. Nat Methods.

[29] TMHMM Server v. 2.0. http://www.cbs.dtu.dk/services/TMHMM/. Accessed Aug 2017.

[30] Grissa I, Vergnaud G, Pourcel C (2007). CRISPRFinder: a web tool to identify clustered regularly interspaced short palindromic repeats. Nucleic Acids Res.

[31] Tatusov RL, Fedorova ND, Jackson JD, Jacobs AR, Kiryutin B, Koonin EV, Krylov DM, Mazumder R, Mekhedov SL, Nikolskaya AN (2003). The COG database: an updated version includes eukaryotes. BMC Bioinformatics.

[32] Fraser CM, Gocayne JD, White O, Adams MD, Clayton RA, Fleischmann RD, Bult CJ, Kerlavage AR, Sutton G, Kelley JM (1995). The minimal gene complement of mycoplasma genitalium. Science.

[33] Citti C, Blanchard A (2013). Mycoplasmas and their host: emerging and re-emerging minimal pathogens. Trends Microbiol.

[34] Do CB, Mahabhashyam MS, Brudno M, Batzoglou S (2005). ProbCons: probabilistic consistency-based multiple sequence alignment. Genome Res.

[35] Browning GF, Noormohammadi AH, Markham PF. Identification and characterization of virulence genes in mycoplasmas. In: Browning GF, Citti C, editors. Mollicutes: molecular biology and pathogenesis. Norfolk: Caister Academic Press; 2014. p. 77–90.

[36] Cassell GH (1982). The pathogenic potential of mycoplasmas: mycoplasma pulmonis as a model. Rev Infect Dis.

[37] Uniprot Consortium (2015). UniProt: a hub for protein information. Nucleic Acids Res.

[38] Woese CR, Kandler O, Wheelis ML (1990). Towards a natural system of organisms: proposal for the domains archaea, Bacteria, and Eucarya. Proc Natl Acad Sci U S A.

[39] Vos P, Garrity G, Jones D, Krieg NR, Ludwig W, Rainey FA, Schleifer K-H, Whitman W. Bergey’s manual of systematic bacteriology: volume 3: the Firmicutes. 2nd ed. New York: Springer-Verlag; 2011.

[40] Whitcomb R, Tully J, Bové J, Bradbury J, Christiansen G, Kahane I, Kirkpatrich B, Laigret F, Leach R, Neimank H (1995). Revised minimum standards for description of new species of the class Mollicutes (division Tenericutes). J Syst Bacteriol.

[41] Edward DGF, Freundt E (1973). Type strains of species of the order Mycoplasmatales, including designation of neotypes for mycoplasma mycoides subsp. mycoides, mycoplasma agalactiae subsp. agalactiae, and mycoplasma arthritidis. Int J Syst Evol Microbiol.

[42] Freundt E (1955). The classification of the pleuropneumonia group of organisms (Borrelomycetales). Int J Syst Evol Microbiol.

[43] American Type Culture Collection Catalog. https://www.atcc.org. Accessed Oct 2017.

[44] Ashburner M, Ball CA, Blake JA, Botstein D, Butler H, Cherry JM, Davis AP, Dolinski K, Dwight SS, Eppig JT (2000). Gene ontology: tool for the unification of biology. The gene ontology consortium. Nat Genet.

[45] Darzentas N (2010). Circoletto: visualizing sequence similarity with Circos. Bioinformatics.

[46] Krzywinski M, Schein J, Birol I, Connors J, Gascoyne R, Horsman D, Jones SJ, Marra MA (2009). Circos: an information aesthetic for comparative genomics. Genome Res.

